# Jet Electrochemical Micromachining of Micro-Grooves with Conductive-Masked Porous Cathode

**DOI:** 10.3390/mi11060557

**Published:** 2020-05-30

**Authors:** Guochao Fan, Xiaolei Chen, Krishna Kumar Saxena, Jiangwen Liu, Zhongning Guo

**Affiliations:** 1 School of Electromechanical Engineering, Guangdong University of Technology, Guangzhou 510016, China; gcfangdut@163.com (G.F.); znguo@gdut.edu.cn (Z.G.); 2 Department of Mechanical Engineering, KU Leuven, 3001 Leuven, Belgium; krishna.saxena@kuleuven.be

**Keywords:** micro-groove, electrochemical machining, porous cathode, conductive mask, machining localization, dimensional uniformity

## Abstract

Surface structures with micro-grooves have been reported to be an effective way for improving the performance of metallic components. Through-mask electrochemical micromachining (TMEMM) is a promising process for fabricating micro-grooves. Due to the isotropic nature of metal dissolution, the dissolution of a workpiece occurs both along the width and depth. Overcut is generated inevitably with increasing depth, which makes it difficult to enhance machining localization. In this paper, a method of electrochemical machining using a conductive masked porous cathode and jet electrolyte supply is proposed to generate micro-grooves with high machining localization. In this configuration, the conductive mask is directly attached to the workpiece, thereby replacing the traditional insulated mask. This helps in achieving a reduction in overcut and an improvement in machining localization. Moreover, a metallic nozzle is introduced to supply a jetted electrolyte in the machining region with enhanced mass transfer via a porous cathode. The simulation and experimental results indicate that as compared with an insulated mask, the use of a conductive mask weakens the electric field intensity on both sides of machining region, which is helpful to reduce overcut and enhance machining localization. The effect of electrolyte pressure is investigated for this process configuration, and it has been observed that high electrolyte pressure enhances the mass transfer and improves the machining quality. In addition, as the pulse duty cycle is decreased, the dimensional standard deviation and roughness of the fabricated micro-groove are improved. The results suggest the feasibility and reliability of the proposed method.

## 1. Instruction

Together with the development of modern micromanufacturing technologies, the requirements for multiple product function integration and structure miniaturization are becoming more important. Therefore, with an increasing demand of microstructures in industrial applications, there is a recent surge in the research on micro-scale features, which have received extensive attention from both academia and industry [1,2]. As a typical example of surface texture, micro-grooves are widely used in fuel cells, cutting tools, hydrodynamic bearings and heat transfer. For example, micro-grooves prepared on the bipolar plate of proton exchange membrane fuel cells (PEMFC) have a significant impact on the performance and operation efficiency, thereby serving the purpose of increasing the output power density of fuel cells [3,4]. Micro-grooved textures can be fabricated on the surface of cutting tools, which can improve the cutting performance and service life, since it reduces the contact length between chips and cutters, storing lubricant and slowing tool wear [5,6]. Owing to the positive impact on lubrication performance, hydrodynamic bearings textured with micro-grooves have better stability and anti-wear property [7,8].

In recent years, many scholars have conducted extensive research on the efficient fabrication of micro-groove surface structures. The common methods for the fabrication of micro-grooves include micro milling [9], laser machining [10], and micro-electrical discharge machining [11], etc. The application of micro-machining is limited by the hardness of the workpiece, tool strength at diameters < 100 µm, tool wear, and tool vibration, which affects the surface quality. Laser processing can make the workpiece surface prone to severe deterioration layers and micro-cracks, and it is difficult to guarantee the machining accuracy of dimension. Micro-electrical discharge machining suffers from the heat-affected zone around the metallographic structure, electrode wear, and machining cost. Compared with these methods, electrochemical machining (ECM) is more suitable in the micromachining domain for the manufacturing of micro-profiles, thin-walled parts, and other difficult machining process. There are unique advantages of ECM over other micro-machining techniques, such as no heat-affected zone, no tool wear, independent of workpiece hardness, and high surface finish, which is a contactless process for metallic material dissolution [12,13].

Jet electrochemical machining (JEM) sprays high-speed electrolyte from the metal nozzle to the surface of the workpiece, which helps to generate deep micro-grooves, since the velocity of the electrolyte and the mass transfer effect of the machining process are improved. Natsu et al. [14] developed an algorithm to control the scanning speed of the nozzle by superimposition and generated micro-groove arrays on the plane and the cylindrical surface. Hackert-Oschätzchen et al. [15] applied the JEM process to fabricate microchannels with a width of 200 μm and a depth of 60 μm by controlling the trajectory of nozzle with inner diameter of 100 μm. Mitchell-Smith et al. [16,17] explored the difference in groove profile and resultant surface finish by changing the inclination angle of the nozzle. However, JEM always suffers from stray corrosion at the edges of micro-grooves and has limitations in micromanufacturing due to hydraulic jump phenomenon and difficulty in fabricating suitable nozzles with small inner diameter [18]. Through-mask electrochemical micromachining (TMEMM) is also an effective approach to fabricate surface microstructures. In the standard TMEMM, the surface of each workpiece is coated with a patterned mask prepared by the lithography process. Based on the electrochemical reaction, the exposed regions of the workpiece surface are dissolved [19]. Zhou et al. [20] employed the TMEMM process to generate micro-grooves ranging 148.5 to 258.6 μm in width and 17.6 to 58.0 μm in depth on the surface of phosphor bronze alloy. They used a mask with a 110 μm wide micro-slit and studied the effect of different machining parameters on the dimensions and morphology of micro-grooves. Overcut in TMEMM is generated inevitably due to the isotropic nature of metal dissolution. It should be noted that the photoresist on the workpiece surface needs to be removed after TMEMM, and therefore it is not reusable. Zhu et al. [21] developed a modified TMEMM process, which replaced the photoresist layer with a re-usable mask and reduced the processing cost. Qu et al. [22] proposed a modified microscale pattern transfer by employing a re-usable cathodic tool carrying the dry-film mask. Wang et al. [23] explored the applicability of electrochemical pattern transfer machining (ECPTM) to fabricate micro-grooves and performed a simulation analysis. It is worth pointing out that ECPTM also suffers from severe stray corrosion on both sides of the micro-groove due to the unprotected surface of the workpiece. Zhang et al. [24] developed a sandwich-like electrochemical micromachining (SLEMM) technology for reducing overcut and improving the dimensional uniformity of micro-dimples by enabling uniform distribution of the electric field in the machining region. However, the generation of bubbles and accumulation of insoluble products in the machining region suppress the dissolution of the workpiece.

In order to evacuate bubbles and some insoluble electrolytic products as well as optimize the cathode structure of conventional SLEMM, Zhang et al. [25] proposed a process scheme of the open reaction unit by using a porous cathode. Ming et al. [26] analyzed and verified the electric field distribution characteristics of the above method and fabricated micro-dimples arrays of dimensional uniformity on the cylindrical workpiece surface. Stray corrosion is an unavoidable phenomenon in TMEMM. The auxiliary anode was proposed to reduce the marginal effect of the electric field on the workpiece, thereby improving the machining accuracy of the microstructure [27]. Chen et al. [28] developed a method of JEM with a conductive mask to generate micro-dimples, which evidently reduced the overcut and enhanced the machining localization.

**Figure 1:**
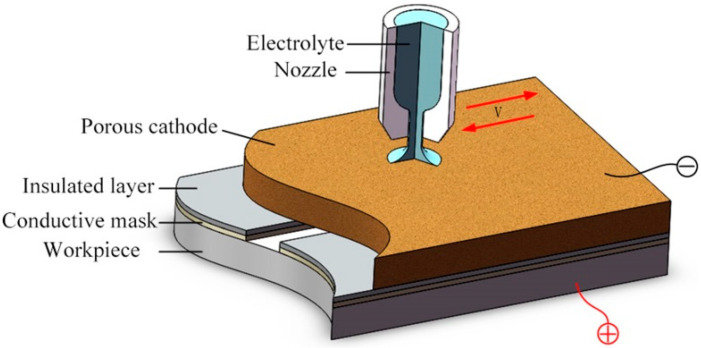
The schematic diagram of jet machining with a conductive masked porous cathode.

The literature has shown the impact of processing technology on the fabrication of microstructures. For the electrochemical machining of micro-grooves, the key to improve the machining quality is to enhance the mass transfer effect in the machining region and reduce overcut to heighten the localization. In previous research [29], we have proposed a method of electrochemical machining of micro-grooves with masked porous cathode, the influence of different flow modes on the machining process was investigated, and a jet flow mode was optimized at last, which could improve the machining efficiency and dimensional uniformity. However, the undercut of micro groove is unavoidable due to the isotropy of material when the traditional insulated mask was used, which reduced the machining localization. In this paper, a conductive masked porous cathode with jet electrolyte supply is proposed (Figure 1) for the fabrication of micro-grooves. The conductive mask is introduced to be directly and firmly attached to the workpiece, replacing the original insulated mask, so that the conductive mask had the same potential as the workpiece. This will weaken the electric field intensity on both sides of the micro-grooves, thereby achieving the purpose of reducing overcut. Moreover, a metallic nozzle is used to increase the flow rate of the electrolyte in the machining region and enhance mass transfer. In this paper, the distribution of the electric field in the machining region is analyzed by establishing a simulation model with different masks, and the evolution processes of the micro-groove profile is predicted as well. The feasibility of the method is reported. Combining with the experimental results, the effect of electrolyte pressure and pulse duty cycle on the fabrication of micro-grooves are analyzed.

## 2. Description of the Method and Numerical Simulation

### 2.1. Description of the Method

Due to relatively larger machining gap in the conventional TMEMM, the mass transfer is promoted. On the other hand, by minimizing the inter-electrode gap, the electric field distribution between the electrodes is uniformly distributed, and the dimensional uniformity is improved. Figure 1 shows a schematic diagram of the jet machining of micro-grooves by using a conductive masked porous cathode. One side of the insulated layer is attached with the porous metal, while the other side is bonded to the conductive mask as a whole, which is covered on the workpiece, and thus the machining region is restrained in a closed unit. The porous metal is used as a cathode and mass transport medium. The columnar pressurized electrolyte is ejected toward the porous cathode from the nozzle, which can provide a high electrolyte velocity to infiltrate into the whole machining region through the porous cathode. A micro-groove can be generated when sufficient voltage is applied between the porous cathode and workpiece. The processing condition in the whole machining region can be improved by controlling the reciprocating motion of the nozzle, which is helpful for the removal of the electrolytic product as well as for the renewal of the electrolytes. After processing, the porous cathode and mask can be re-used, and it could reduce the processing cost as well as improve the machining efficiency.

### 2.2. Numerical Simulation

In this study, both the porous cathode and the conductive mask were introduced. For comparative analysis, the electric field distribution in the machining region was investigated with both insulated and conductive masks by establishing a mathematical simulation model, and the evolved shape of the micro-groove was predicted.

#### 2.2.1. Model Building

**Figure 2:**
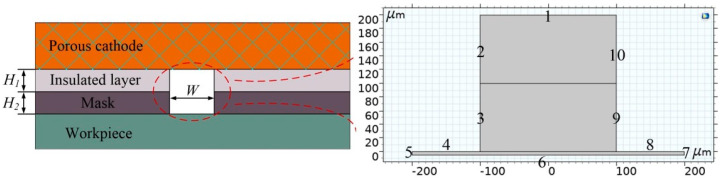
The 2D model of electrochemical machining (ECM) with a masked porous cathode.

Since only small potential gradients are expected in the metallic electrodes due to the high conductivities, the electrode domains are not included in the model. The insulated layer is electrochemically inert, and hence it is not included either. The only modeled domain is the electrolyte. By simplifying the process model, the 2D electric field simulation model of the machining region is established in the inter-electrode gap, as shown in Figure 2. *H*_1_ and *H*_2_ denote the thickness of insulated layer and mask, respectively. *W* is the width of the micro-slot of the mask. Considering that the anode voltage during actual processing is distributed over the entire workpiece, a small virtual area with a length of 400 μm and height of 5 μm is added at anode end, which also makes boundary 6 move properly without mesh distortion.

According to electric field theory, the electric potential *φ* is governed by Laplace’s equation [30]:

(1) $$\nabla^{2}\varphi =0.$$

According to Ohm’s law, the current density, $\vec{i}$, can be evaluated as follows:

(2) $$\vec{i}=\sigma \vec{E}$$

where *σ* is the electrolyte conductivity and $\vec{E}$ is the electric field intensity on the workpiece. 

According to Faraday’s Law, the rate of material dissolution, $\vec{v}$, can be expressed as follows:

(3) $$\vec{v}=\eta \omega \vec{i}=\eta \omega \sigma \vec{E}$$

where *η* represents the current efficiency and ω represents the volumetric electrochemical equivalent of the material.

In this study, the current efficiency can be expressed as follows [31]:

(4) $$\eta (i)=\frac{0.85}{\left(1+e^{(10-i)/6}\right)}-0.1.$$

To simplify the model of the electric field established in the machining process, the following assumptions are made:

1. The reaction surface of porous cathode is considered as a solid flat surface, ignoring the internal porous structure.
2. The potential loss due to the electrochemical reaction is ignored, and Ohm’s law and charge balance are used to calculate the current in the electrolyte and electrode.
3. The conductivity of the electrolyte, *σ*, is constant.
4. The concentration gradient in the bulk electrolyte is negligible.

In the model, the primary current distribution is used to calculate the electric field intensity. The domain of computational interest in the model is the zone of electrolyte. Boundary 1, whose external potential is set to 0 V (ground), is the cathode reaction surface (porous cathode surface). Boundary 6 is the anode reaction surface (workpiece surface), which is dissolved during the electrochemical reaction process, and its external potential is set to *U*. With a conductive mask, Boundaries 3–4 and 8–9 are set to the electric potential of *U*. Correspondingly, they are set to electric insulation with an insulated mask, and the other undefined boundaries are set as electric insulation.

By coupling the primary current distribution and deformed geometry module, the process of material dissolution was simulated. Combining Equations (3) and (4), deformation of Boundary 6 can be obtained. Then, the evolved shape of the micro-groove profile is described. In this model, the boundaries are set as follows:

(5) $$\left\{ \begin{array}{l} \frac{d_{x}}{d_{t}}|\Gamma_{2,3,5,7,9,10}=0\\ \frac{d_{y}}{d_{t}}|\Gamma_{1,4,8}=0\\ \frac{\partial (x,y)}{\partial_{n}\partial_{t}}|\Gamma_{6}=v_{n} \end{array}\right.$$

where *v_n_* represents the normal anodic dissolution rate on the workpiece in Boundary 6.

The parameters used in the simulation are listed in Table 1. The model is solved on a commercial finite element platform COMSOL^®^ Multiphysics software version 5.4.

#### 2.2.2. Simulation Results

**Figure 3:**
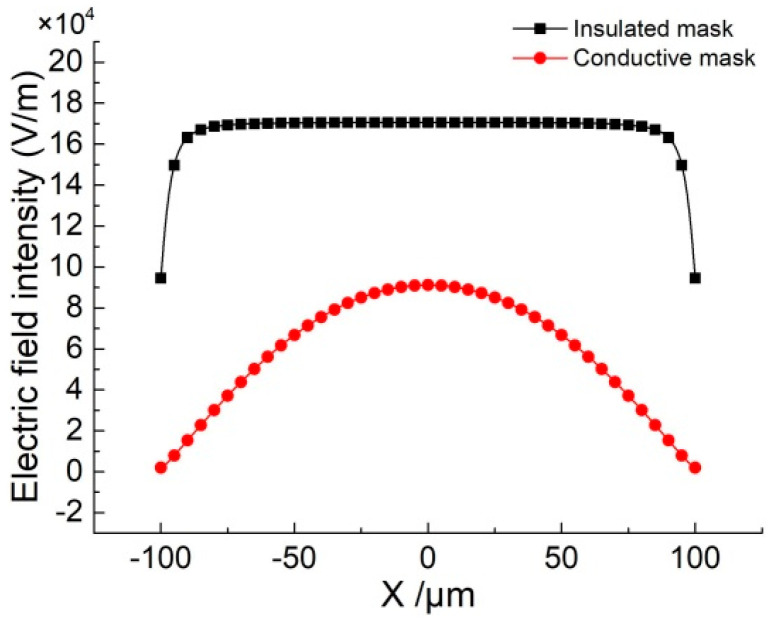
Electric field intensity on the workpiece surface using different masks.

The initial electric field distribution on the exposed workpiece surface simulated using conductive and insulated masks is shown in Figure 3. It can be seen that the electric field intensity using an insulated mask is greater than that obtained with a conductive mask at each position. The electric field intensity using the conductive mask is gradually increased from the edge to the center in the machining region. Correspondingly, with the insulated mask, the electric field intensity is quickly transitioned from the minimum value at the edge to the maximum constant value in the middle region. By observing the magnitude of the electric field intensity value, it can be inferred that the dissolution rate of the material is proportional to the electric field intensity according to Equations (2)–(4). In process terms, this means that more material is dissolved at the region toward edges with the insulated mask, while this is not the case when the conductive mask is used.

**Figure 4:**
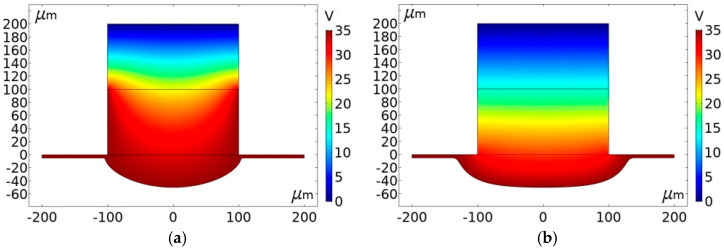
Simulation results using different masks. (**a**) Using the conductive mask; (**b**) Using the insulated mask.

Figure 4 shows the simulation results for grooves generated with the same depth of 45 μm using conductive and insulated masks, respectively. It can be seen that while using an insulated mask, the groove is much wider than those obtained with a conductive mask. This is a strong indication that the machining localization can be enhanced with the conductive mask.

**Figure 5:**
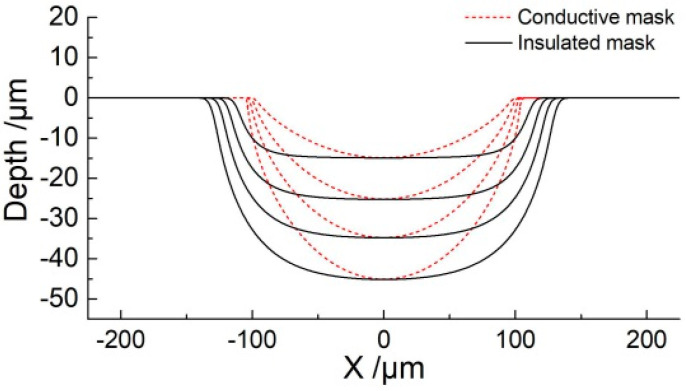
Cross-section profiles of micro-grooves with different masks.

In order to further analyze the differences in the micro-grooves fabricated using different masks, simulations were conducted at different depths to compare the change in width. Figure 5 shows cross-section profiles of micro-grooves generated at different depths (15 μm, 25 μm, 35 μm, and 45 μm). The profiles also reflect the evolution process of micro-grooves with a conductive mask and insulated mask. As the depth of the micro-grooves fabricated with an insulated mask is increased, the width also increases. Correspondingly, with a conductive mask, there was only a small increase in the width, even at higher depths. Moreover, at the same depth, the width of micro-grooves generated with a conductive mask was smaller than that of using an insulated mask. The phenomenon can be explained as follows.

The electric field is confined to a closed machining region with the proposed method. For the use of the insulated mask, the electric field is formed directly by a porous cathode and workpiece anode. It can be seen from Figure 3 that the electric field intensity is larger compared with the conductive mask. According to Equation (3), although the electric field intensity at the center of the machining region is much greater than the edges, the material of the width direction is also dissolved to a certain extent with the increase of depth. Correspondingly, for the use of the conductive mask, the conductive mask has the same potential as the workpiece anode, so that the electric field also is formed by a conductive mask and porous cathode, which weakens the electric field intensity on the workpiece edge surface of the machining region. Furthermore, the width growth range is even smaller compared with the depth increasing. Through comparative analysis, the use of a conductive mask is more helpful to reduce the overcutting and improve the machining localization.

## 3. Experimental

**Figure 6:**
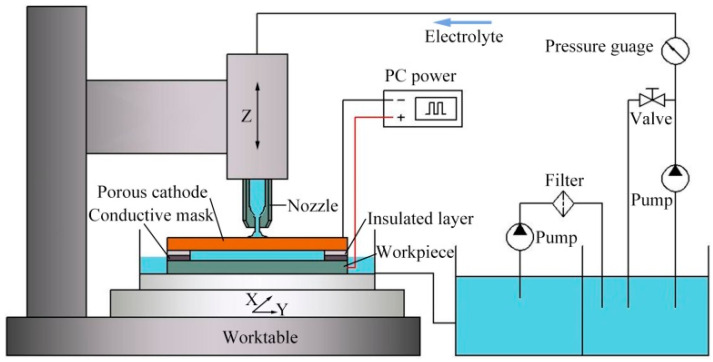
Schematic of an experimental system.

The developed experimental system is schematically shown in Figure 6. It consists of a servo-control feed unit, platform control unit, electrolyte circulation system, and power supply. The conductive porous cathode and workpiece were fixed on the X/Y stage by a special fixture. The nozzle was clamped on the Z-axis by the holder. The motion route and speed of the nozzle were controlled along the position of the through-mask above the porous cathode. The electrolyte circulating system could realize the reuse of an electrolyte and ensure the refreshing of gap conditions through the recirculation of an electrolyte. In the end, the micro-groove could be fabricated by applying a sufficient voltage.

**Figure 7:**
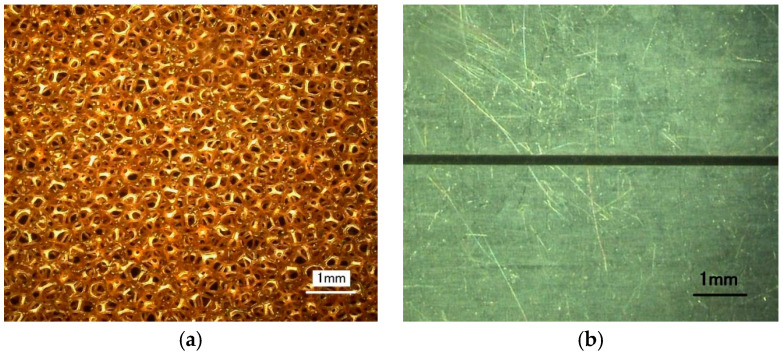
The image of conductive masked porous cathode. (**a**) Porous cathode; (**b**) Conductive mask.

In this experiment, a porous copper plate was introduced as a porous cathode (Figure 7a), and a platinum sheet with a thickness of 100 μm was employed as the conductive mask, which showed a high chemical inertness and could not be dissolved during machining. The micro-slot with a length of 20 mm and a width of 200 μm in the mask was prepared by a micro-electrical discharge process (shown in Figure 7b). The flexible insulated layer was made of epoxy resin with a thickness of 100 μm. During the electrochemical machining, the nozzle was reciprocated once, and a nozzle with an inner diameter of 2 mm was utilized. The nozzle is moved 1 mm away from the porous cathode. The main experiment parameters are listed in Table 2. In our previous research, we have found that increasing the reciprocating motion number (increasing the moving speed of nozzle) could reduce the machining roughness in high machining voltage (high current density), but it has little influence on the machining roughness in low voltage (low current density), because there was less electrolytic product [29]. According to the simulation result in Figure 3, it is found that with a conductive mask, the electric field intensity on the workpiece is quite lower than that with an insulated mask, which means a low current density on workpiece during machining. According to the previous research result, the influence of the reciprocating motion number on the roughness would not be obvious due to the low current density, and our pre-experiments have also proved it, thus one reciprocating motion of a nozzle was used in this study.

A hall current sensor was used to detect the current signal, and a data acquisition card (NI 9222, Austin, TX, USA) was introduced to record current with a sampling frequency of 100 kHz. In order to analyze the repeatability of fabrication of a micro-groove with diffident parameters, each set of experimental parameters was repeated twice. Seven points were measured for each micro-groove, the average value of the two micro-grooves was obtained, and the standard deviation (SD) was used to evaluate the dimensional uniformity of a micro-groove. The profiles and bottom roughness of micro-grooves were measured using a confocal laser-scanning microscope (CLSM, Olympus LEXT OLS4000, Tokyo, Japan).

## 4. Results and Discussion

### 4.1. Comparison of Micro-Grooves Generated with Conductive and Insulated Mask

For the purpose of comparing the difference in the fabrication of micro-grooves with different masks, the experiments were conducted with pulse power supply setting a pulse duty cycle of 20% and pulse frequency of 1 kHz. Among them, the applied voltage of 10 V and machining time of 10 s with the insulated mask were adopted, and the applied voltage of 35 V and machining time of 20 s with the conductive mask were employed. It should be pointed out that the use of a conductive mask weakens the electric field intensity in the machining region, requiring a larger voltage during processing. The insulated mask and conductive mask with thicknesses of 100 μm were designed with same structure. The width and length of the micro-slot in the masks were 200 μm and 20 mm, respectively.

**Figure 8:**
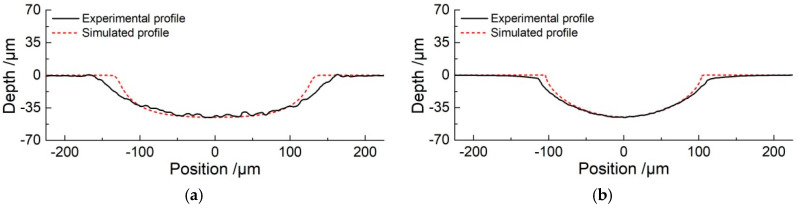
Profiles of micro-grooves in same depth fabricated with different masks. (**a**) Using the insulated mask (*P_in_* = 0.8 MPa, *U* = 10 V, *ε* = 20%, *f* = 1 kHz, *t*_on_ = 10 s); (**b**) Using the conductive mask (*P_in_* = 0.8 MPa, *U* = 35 V, *ε* = 20%, *f* = 1 kHz, *t*_on_ = 20 s).

Figure 8 shows profiles of fabricated micro-grooves with same depth of 45 μm using insulated and conductive masks. It can be seen that the width of the micro-groove was about 319.4 μm by using an insulated mask (Figure 8a). However, when a conductive mask was introduced, the width of the micro-groove was about 227.3 μm (Figure 8b). This is an indication that overcut is significantly improved with a conductive mask as compared to using the insulated mask. The etch factor, which is defined as depth/(width of groove—width of mask), increases from 0.75 to 3.3, and it reflects that the machining localization of the micro-groove is enhanced. Although the experimental profiles show good agreement with the simulation results, the undercut of its width is greater than the simulation result, which reduces the machining accuracy. The main reason for this observation can be that the mask cannot be adhered firmly to the workpiece, which causes the electrolyte to penetrate into the gap between the mask and the workpiece, resulting in overcut along the width.

It is clear from the above discussions that the undercut using the conductive mask is smaller than that using the insulating mask, showing that the conductive mask can reduce the electric field intensity on both sides of the machining region. On the other hand, the use of a conductive mask requires a higher voltage and a longer time, which reflects that the energy consumption was increased. The following experiments were performed to study the effect of electrochemical machining of micro-grooves using a conductive masked porous cathode and jetted electrolyte.

### 4.2. Micro-Grooves Generated with Different Electrolyte Pressures

**Figure 9:**
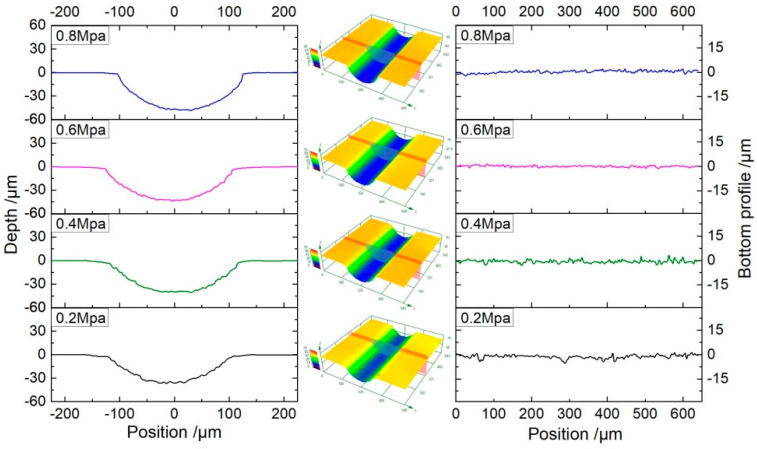
The profiles of micro-grooves generated with different electrolyte pressures (*U* = 35 V, *ε* = 20%, *f* = 1 kHz, *t*_on_ = 20 s).

In order to investigate the profile of micro-grooves generated at different electrolyte pressure, the experiments were prepared with a PC voltage of 35 V, pulse duty cycle of 20%, pulse frequency of 1 kHz, and machining time of 20 s. Figure 9 shows the cross-section profiles, the 3D profiles, and the bottom profiles of the micro-grooves. It can be seen that the dimensional uniformity of the micro-groove of jet machining using the conductive masked porous cathode is good. As the electrolyte pressure is increased, profiles of micro-grooves become smoother.

**Figure 10:**
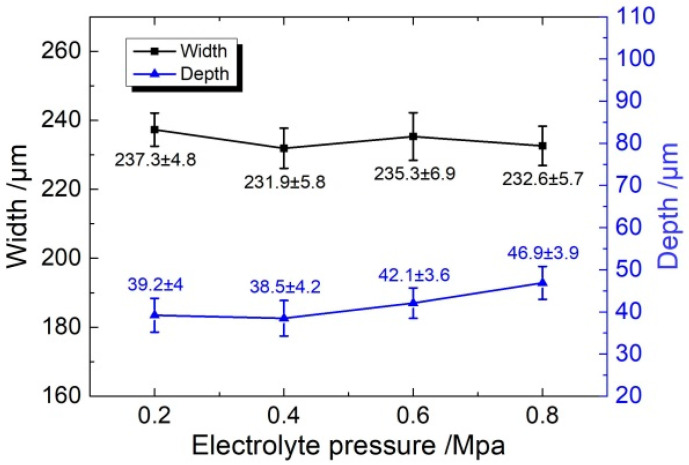
The dimension of micro-grooves generated with different electrolyte pressures (*U* = 35 V, *ε* = 20%, *f* = 1 kHz, *t*_on_ = 20 s).

**Figure 11:**
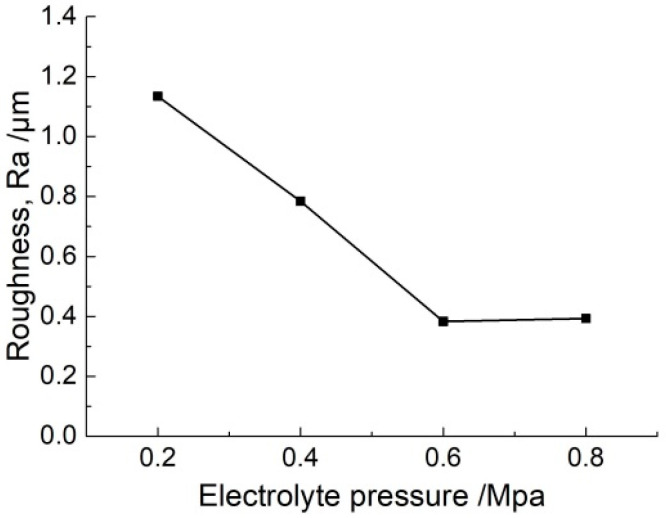
The roughness of micro-grooves generated with different electrolyte pressures (*U* = 35 V, *ε* = 20%, *f* = 1 kHz, *t*_on_ = 20 s).

Figure 10 and Figure 11 show the dimension and roughness of micro-grooves fabricated at different electrolyte pressures, respectively. When the electrolyte pressure is increased from 0.2 MPa to 0.8 MPa, the width increases from 237.3 ± 4.8 μm to 232.6 ± 5.7 μm (mean ± SD), the depth increases from 39.2 ± 4 μm to 46.9 ± 3.9 μm, and the roughness (Ra) decreases from 1.13 μm to 0.39 μm. It shows that with an increase in electrolyte pressure, the electrolyte pressure helps to make the conductive masked porous cathode adhere better to the workpiece. This reduces the penetration of electrolyte between the mask, and the workpiece and closed electric field is maintained. Consequently, grooves with smaller width and higher depth are obtained, and the standard deviation is maintained at a smaller value. In the case of low electrolyte pressure of 0.2 MPa and 0.4 MPa, the mass transfer of electrolyte became relatively weakened due to the infiltration of insoluble reaction by-products as well as gas bubbles into the porous cathode. This suppressed further electrochemical machining resulting in a poor flat uniformity of the bottom profile along the length direction. Compared with electrolyte pressure of 0.2 MPa and 0.4 MPa, a high electrolyte velocity could be provided around the machining region in the applied electrolyte pressure of 0.6 MPa and 0.8 MPa, which was helpful for refreshing the electrolyte and the evacuation of the insoluble products, bubbles, and heat. The electrochemical machining environment is optimized by reciprocating the nozzle, which could increase the stability of the machining process. Therefore, the prepared micro-groove shows small roughness and high machining quality. The electrolyte pressure was selected to 0.8 MPa in the following experiments.

### 4.3. Micro-Grooves Generated with Different Pulse Duty Cycles

Besides the electrolyte pressure, the pulse duty cycle also has a significant effect on the fabrication of micro-grooves. The pulse current is helpful to improve the machining accuracy of ECM and to refresh the electrolyte during the pulse-off time. In the above study, the pulse duty cycle of 20% was utilized. In order to investigate the influence of the pulse duty cycle on the machining process, experiments with different pulse duty cycles were conducted at a voltage of 35 V.

**Figure 12:**
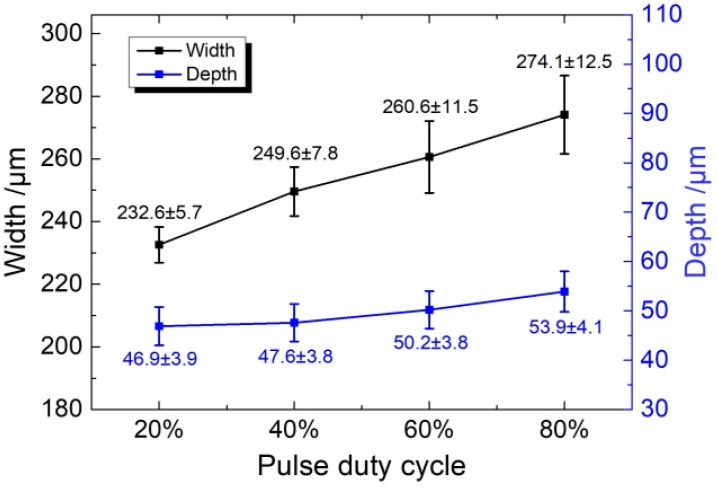
The dimension of micro-grooves generated with different pulse duty cycles (*P_in_* = 0.8 MPa, *U* = 35 V, *f* = 1 kHz, *t*_on_ = 20 s).

Figure 12 shows the dimensions of the micro-grooves fabricated with different pulse duty cycles at a frequency of 1 kHz and for the same machining time of 20 s. It can be seen that there is an obvious change in the width of the micro-grooves with an increasing pulse duty cycle. The width increases from 232.6 ± 5.7 μm to 274.1 ± 12.5 μm when the pulse duty cycle increases from 20% to 80%. On the other hand, the depth of the micro-groove showed a slight change from 46.9 ± 3.9 μm to 53.9 ± 4.1 μm.

**Figure 13:**
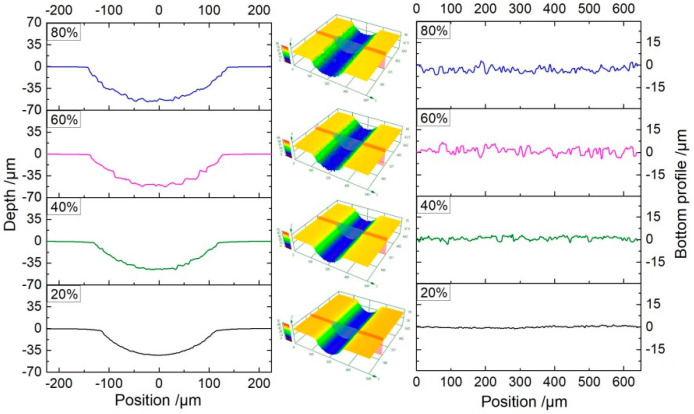
The profile of micro-grooves generated with different pulse duty cycles (*P_in_* = 0.8 MPa, *U* = 35 V, *f* = 1 kHz, *t*_on_ = 20 s).

**Figure 14:**
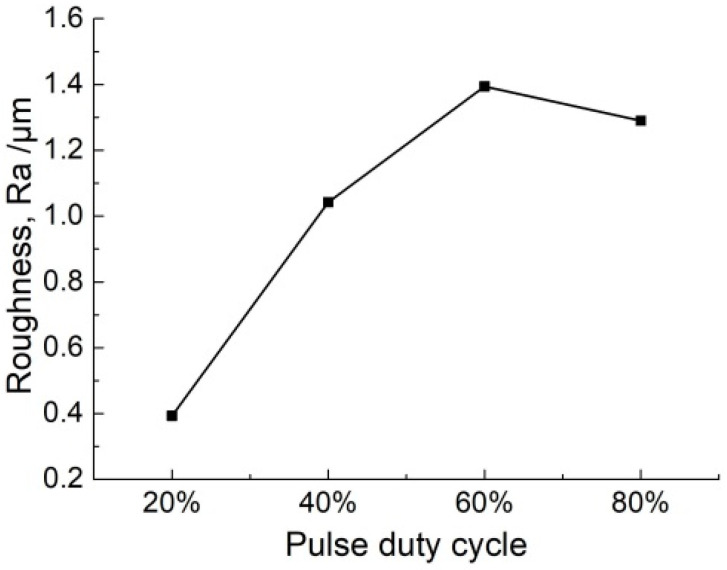
The roughness of micro-grooves with different pulse duty cycles (*P_in_* = 0.8 MPa, *U* = 35 V, *f* = 1 kHz, *t*_on_ = 20 s).

As we know, the electrochemical reactions cause heat generated in the bulk of the electrolyte (Joule heating) during machining. Especially with a conductive mask, there is a larger reaction area than that in insulated mask, and there would be a large amount of heat generated. This heat increases the electrolyte temperature, resulting in a further increase of the electrolyte conductivity. In the pulse current, the pulse-off time is used to flush the heat and by-products out of the machining gap as well as renew the electrolyte. Consequently, the influence of heat on the machining process is reduced. When the pulse duty cycle is increased, the pulse-off time is reduced. As a result, the heat transport process in the machining gap is weakened, which increases the electrolyte conductivity. According to Equation (3), the material removal rate is increased with a high pulse duty cycle. Figure 12 shows that with the increasing pulse duty cycle, both the width and depth are enlarged. On the other hand, the decrease of pulse-off time weakens the removal of electrolytic products in the gap. As a result, the accumulation of electrolytic products at the bottom of the micro-groove is increased, and it hinders the material dissolution at the bottom of the micro-groove. That is why the increase of the material removal rate is more in the lateral direction (width) as compared to the axial direction (depth). Meanwhile, the accumulation of electrolytic products led to a non-uniform dissolution of material in both width and depth, and it not only increased the error bar, but also reduced the surface quality. Figure 13 and Figure 14 show the profiles and roughness of micro-grooves fabricated with different pulse duty cycles, respectively. It can be observed that as the duty cycle is reduced from 80% to 20%, the surface roughness (Ra) reduces to 0.393 μm. The localization and surface quality of micro-grooves is improved with a low pulse duty cycle. Therefore, the pulse duty cycle of 20% is found to be more suitable for the preparation of micro-grooves by this process scheme.

## 5. Conclusions

This paper proposed a method of electrochemical machining of micro-grooves using a conductive masked porous cathode and jet electrolyte supply. From the simulation and experimental results, the concluding remarks can be summarized as follows.

1. The simulation results indicated that the use of a conductive mask reduced the electric field intensity on both sides of the micro-groove and achieved the purpose of reduction in overcut.
2. On comparing the results with an insulated mask and a conductive mask to fabricate micro-grooves with the same depth of 45 μm, it was observed that the etch factor was increased from 0.75 (insulated mask) to 3.3 (conductive mask), which showed that the machining localization of micro-grooves was enhanced.
3. In this process scheme, a high electrolyte pressure was favorable for the renewal of the electrolyte and enhanced mass transfer during processing, which improved the machining quality and dimensional uniformity of the micro-grooves.
4. The pulse duty cycle has an important effect on the machining localization. A low pulse duty cycle of 20% could obtain micro-grooves with better machining localization and surface quality.

## Figures and Tables

**Table 1:** The parameters set for the simulation.

Parameters	Value
Thickness of the insulated layer, *H*_1_	100 μm
Thickness of the mask, *H*_2_	100 μm
Width of the micro-slot, *W*	200 μm
Electrolyte conductivity, *σ*	12 S/m
Volumetric electrochemical equivalent, *ω*	0.035 mm^3^/(A·s)
Electric potential, *U*	35 V

**Table 2:** Experimental machining parameters.

Parameters	Value
Electrolyte concentration	12% (wt %), NaNO_3_
Electrolyte temperature, *T*	25 °C
Inner diameter of nozzle, *d*	2 mm
Electrolyte pressure, *P_in_*	0.2, 0.4, 0.6, 0.8 MPa
Thickness of the porous cathode, *T*_1_	3 mm
Porosity of the porous cathode, *ε_p_*	0.95
Thickness of the insulated layer, *T*_2_	100 μm
Thickness of the conductive mask, *T*_3_	100 μm
Length of the micro-slit, *L*	20 mm
Width of the micro-slit, *W*	200 μm
Reciprocating motion number of the nozzle, *N*	1
Applied voltage with the insulated mask, *U*_1_	10 V
Applied voltage with the conductive mask, *U*_2_	35 V
Pulse duty cycle, *ε*	20%, 40%, 60%, 80%
Pulse frequency, *f*	1 kHz
Machining time with the insulated mask (*t*_on_), *t*_1_	10 s
Machining time with the conductive mask (*t*_on_), *t*_2_	20 s
Workpiece material	Stainless steel 304
Metallic nozzle material	Stainless steel 304
